# The SARS-CoV-2 main protease causes mitochondrial dysfunction in a yeast model

**DOI:** 10.1038/s41598-025-11993-w

**Published:** 2025-07-18

**Authors:** Wojciech Grabiński, Anna Kicińska, Karolina Funtowicz, Tomasz Skrzypczak, Andonis Karachitos

**Affiliations:** 1 Department of Bioenergetics, Institute of Molecular Biology and Biotechnology, Faculty of Biology, Adam Mickiewicz University, Poznań, Poland; 2 Center for Advanced Technology, Adam Mickiewicz University, Poznań, Poland

**Keywords:** Viral infection, Energy metabolism

## Abstract

*Saccharomyces cerevisiae* has proven to be an invaluable model organism for studying mitochondrial function owing to its genetic tractability and the high conservation of mitochondrial processes among eukaryotes, including humans. Yeasts are easy to culture and manipulate genetically, which allows rapid generation of mutant strains and detailed dissection of mitochondrial pathways. In addition, the ability of yeasts to survive without functional mitochondria allows the study of mutations that are lethal to organisms that are dependent on aerobic metabolism. Taking advantage of these benefits, we investigated the toxicity of SARS-CoV-2 main protease (Mpro) expression in yeast under conditions that enforce mitochondria-dependent aerobic metabolism. Our results showed that Mpro expression was highly toxic and significantly impaired yeast growth. Pronounced changes in the morphology and mitochondrial function were observed, indicating that mitochondrial pathways are exceptionally sensitive to Mpro activity. These results provide insights that may be relevant for understanding the effects of Mpro in more complex eukaryotic systems.

**Supplementary Information:**

The online version contains supplementary material available at 10.1038/s41598-025-11993-w.

## Introduction

Increasing amounts of data suggest that in cells infected with *Betacoronavirus pandemicum* (SARS-CoV-2), mitochondria are central to understanding COVID-19 pathogenesis. In human and rodent cells multiple mitochondrial changes, both structural and functional, have been found to have far-reaching consequences. These include hijacked mitochondria, reprogrammed metabolism, decreased oxidative phosphorylation (OXPHOS), increased reactive oxygen species (ROS) production, elevated mitochondrial iron levels, loss of mitochondrial integrity, cell death, disturbed autophagy, a reduced mitochondrial antiviral response (MAVS) and a type 1 interferon (IFN) response (see^1–3^). Twenty out of 27 viral proteins have been implicated in these processes and have been shown to interact with host mitochondria^4,5^.

The SARS-CoV-2 main protease (Mpro), also known as 3-chymotrypsin-like protease (3CLpro), is a dimeric cysteine protease that is responsible for the cleavage of the viral polyproteins pp1a and pp1b, which are required for viral replication and transcription^6^. This enzyme shows no homology to human proteases and is thus an interesting drug target. As viral proteases cleave host proteins to hinder host immune responses and promote viral replication, numerous host proteins are predicted, via both computational and experimental methods, to be targeted by Mpro^7^. The human proteins confirmed to be cleaved include RNA polymerase II-associated protein 1 (RPAP1), Interleukin-1 receptor-associated kinase 1 (IRAK-1), nucleotide-binding oligomerization domain-like receptor (NLR), Solute carrier family 25 member 22 (SLC25A22)^7^. Among other processes, Mpro activity has been shown to influence human cell transcription and translation, apoptosis, the DNA damage response, lipid metabolism, vesicle trafficking, and the innate immune response^7,8^.

In our study, we used a newly developed yeast system^9^ to explore the effects of Mpro expression on mitochondria. We showed for the first time that Mpro caused severe deterioration of mitochondrial function, growth arrest on nonfermentable carbon sources and significant respiratory rate inhibition in yeast.

## Results

### The effects of different carbon sources on optimizing the expression of the inducible EGFP gene under the control of the *GAL1* promoter in *S. cerevisiae*

**Fig. 1:**
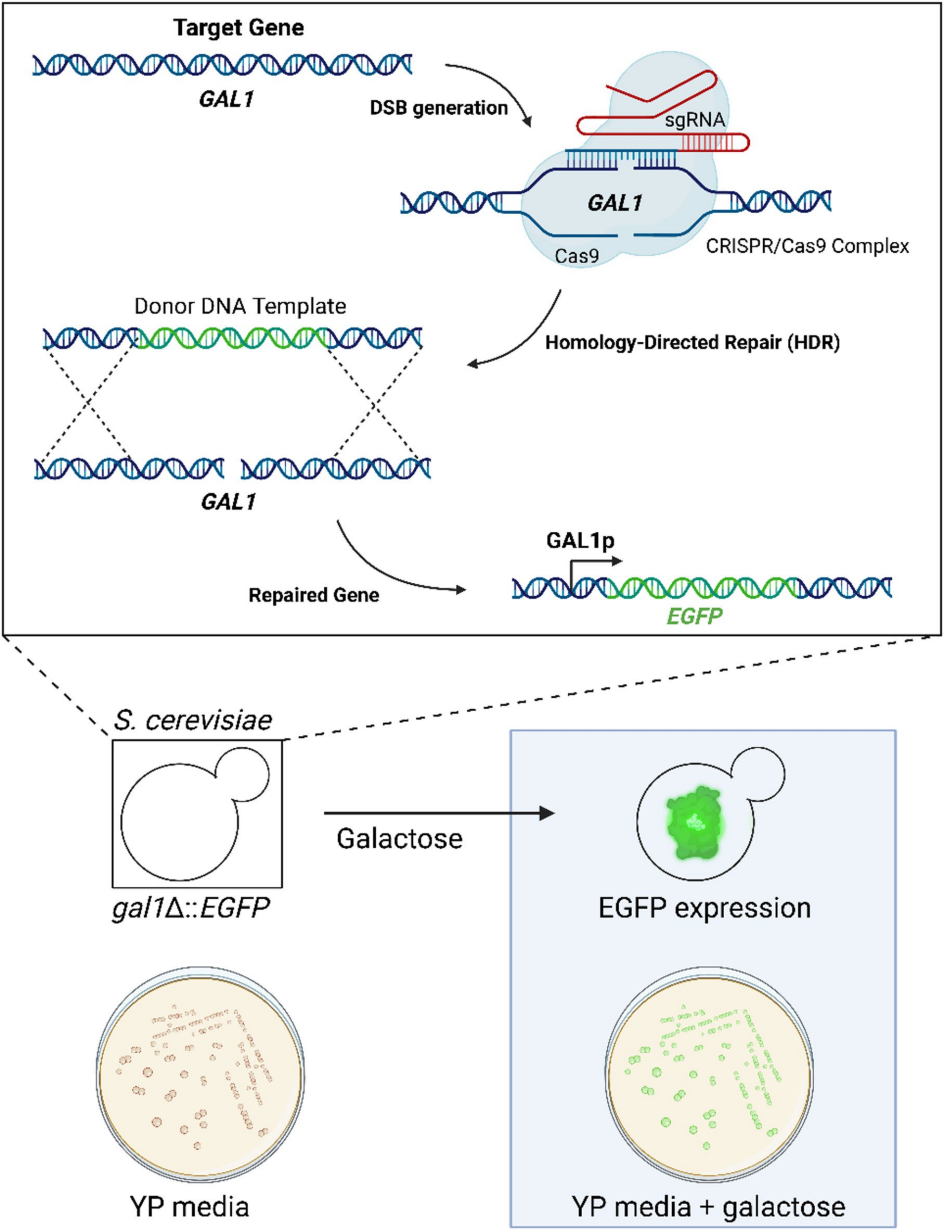
CRISPR/Cas9-mediated knock-in and inducible gene expression under the GAL1 promoter. The *GAL1* gene editing technique used the CRISPR/Cas9 system and a process known as gene repair using the homologous recombination (HDR) mechanism. The initial step involved the identification of the *GAL1* target gene to be modified. The CRISPR/Cas9 complex, which is composed of a Cas9 nuclease and single guide RNA (sgRNA), has the ability to recognize and precisely locate the target site in the DNA, resulting in the formation of a double-strand break (DSB). In the presence of a DNA donor template, which contained homologous sequences and additional elements, such as EGFP, the damaged site was repaired via the HDR mechanism. As a result, the *EGFP* gene was introduced (a knock-in mechanism). This gene was under the control of the inducible *GAL1* promoter. Concurrently, the *GAL1* gene was deleted, enabling comprehensive regulation of the expressed gene through optimized induction conditions. Created in BioRender. Karachitos, A. (2025) https://BioRender.com/c66t872.

A unique platform for gene expression studies in *S. cerevisiae* was developed using CRISPR/Cas9 technology^10^. A stable *EGFP* gene expression system was created by precise genome editing to integrate the *EGFP* reporter gene under the control of the *GAL1* promoter with simultaneous deletion of the *GAL1* gene, as shown in Fig. 1. Under these conditions, galactose metabolism in yeast cells is blocked, and galactose in the culture medium acts solely as an inducer of expression^9^.

The resulting system allows stable and precise induction of gene expression in the presence of galactose, providing a useful platform for further gene regulation studies in a yeast model. Integration of the EGFP reporter gene directly into the native *GAL1* locus, with all its regulatory elements, ensures precise EGFP expression in a natural genomic context. Unlike plasmid-based systems, this system allows the use of rich media, such as YP with a carbon source, thereby avoiding the limitations of synthetic media, which often result in a slow growth and metabolic stress.

**Fig. 2:**
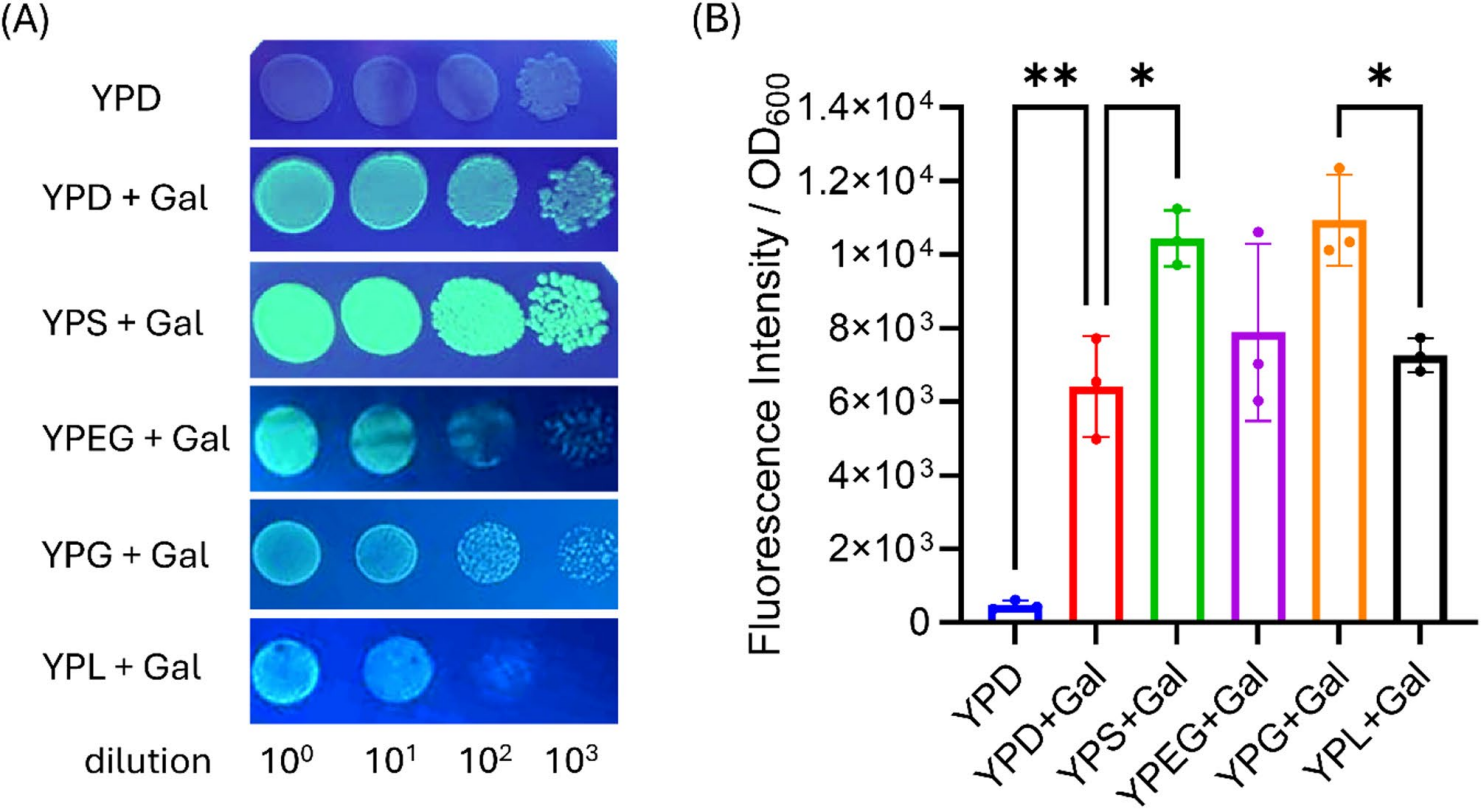
*GAL1* promoter activity depends on the carbon source used in the medium and the presence of an inducing factor. Effect of carbon source on EGFP expression under the *GAL1* promoter. (**A**) Representative plates from a drop assay exposed to UV light using a transilluminator. (**B**) Analysis of the green fluorescence of cells of the EGFP strain grown on different types of media. Statistical analysis was performed using two-way ANOVA. Tukey’s multiple comparisons test was used to compare the means, with significant differences between conditions indicated as follows: *P < 0.05, **P < 0.01. Statistical analysis was based on three biological replicates.

As yeast metabolism is notably modified by the available carbon sources, the system was validated using a whole array of both fermentative and nonfermentative media. EGFP fluorescence was measured to assess *GAL1* promoter activity and expression efficiency (Fig. 2A). Under control conditions (YPD medium without galactose), the fluorescence levels were insignificant, confirming the lack of promoter induction in the presence of glucose as the sole carbon source. In contrast, evident activation of the *GAL1* promoter and an increase in the fluorescence signal were observed in all the tested media supplemented with galactose.

As shown in Fig. 2B, detailed analysis of the quantitative data allowed comparison of the levels of fluorescence in cells growing in media containing various carbon sources (i.e., sucrose, lactate, glycerol, ethanol or a combination of glycerol and ethanol) and galactose. To eliminate the effects of differences in culture growth rates among various media, the data were normalized to the optical density (OD_600_).

The results of the measurements revealed that the highest levels of fluorescence were obtained in YPG (glycerol; 10938 ± 1232) and YPS (sucrose; 10442 ± 763.7). In contrast, in the presence of glycerol and ethanol (YPEG medium), the fluorescence signal was moderate (7888 ± 2413), which may have been due to the simultaneous effects of the two carbon sources on yeast metabolism. The lowest induction of the *GAL1* promoter among the galactose media tested was observed for YPL (lactate; 7267 ± 457.8).

Importantly, clear activation of the *GAL1* promoter was observed even in a medium containing glucose (the strongest catabolic repressor), demonstrating the high flexibility of this expression system. Significant differences between YPD + Gal and YPS + Gal and YPG + Gal conditions were confirmed, highlighting the importance of careful carbon source selection in the design of efficient expression systems. In contrast, no significant differences were found between the YPS + Gal and YPG + Gal media, suggesting similar efficacies of these substrates in the induction of *GAL1* promoter-controlled expression.

The developed *GAL1* promoter-based expression system showed great flexibility in terms of the carbon source used, including glucose, a well-known catabolic repressor. These results clearly indicate that sucrose and glycerol may be good alternatives.

### The SARS-CoV-2 Mpro as an *S. cerevisiae* growth inhibitor under fermentative and respiratory conditions

**Fig. 3:**
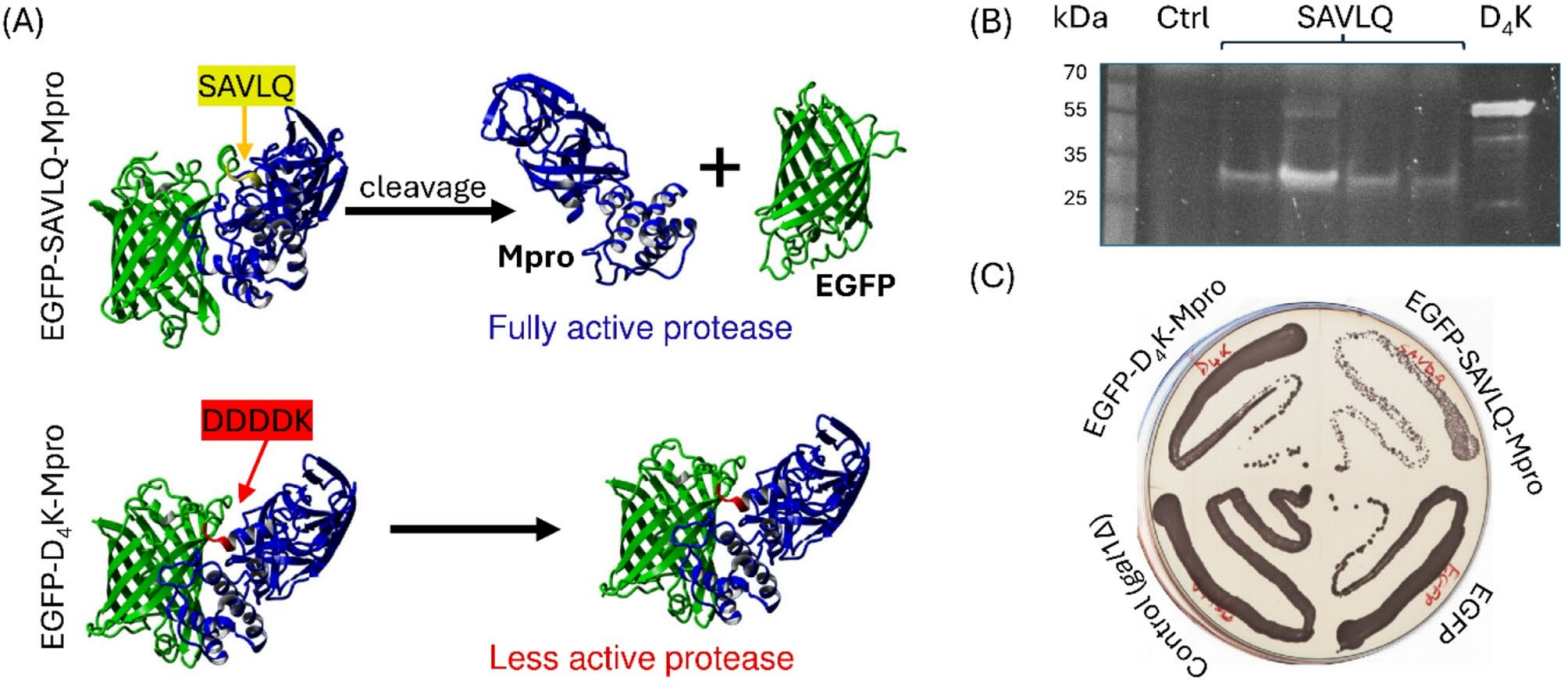
Structures of EGFP fusion proteins with Mpro and their impacts on yeast growth. (**A**) Schematic representation of EGFP-Mpro fusion proteins. The EGFP-SAVLQ-Mpro construct contained a specific sequence between EGFP and Mpro (yellow arrow) that was recognized and autocatalytically cleaved by Mpro, leading to the release of Mpro in its native form, which facilitated its proteolytic activity. The EGFP-D4K-Mpro construct had a linker sequence (red arrow) of the same length between EGFP and Mpro that was not recognized by Mpro, causing the fusion protein to remain intact and significantly reducing its proteolytic activity. (**B**) SDS-PAGE analysis of EGFP fusion proteins expressed in yeast, followed by visualization of EGFP fluorescence. The cleaved and uncleaved forms of the fusion proteins can be distinguished. “Ctrl” denotes the control sample without EGFP expression. (**C**) Growth of yeast strains expressing different constructs on YPS medium supplemented with 1% galactose. The plate shows the following strains in streaks: EGFP-D₄K-Mpro (top left), EGFP-SAVLQ-Mpro (top right), EGFP (bottom right), and a control strain that does not express EGFP (bottom left). Yeast expressing EGFP-SAVLQ-Mpro showed reduced growth due to the toxic effects of active Mpro. In contrast, strains expressing EGFP-D₄K-Mpro, EGFP alone, or the control strain grow normally, indicating the absence or reduced Mpro activity. All 3D structures of the fusion proteins were modeled using ColabFold^34^ version 1.5.5 (https://github.com/sokrypton/ColabFold).

Our expression system was used to assess the influence of the SARS-CoV-2 Mpro on the yeast under different conditions. The gene encoding the EGFP protein was linked to Mpro through two different linkers, SAVLQ and DDDDK (D4K), and both constructs were introduced into yeast as previously described^9^. As shown in Fig. 3A, the two linkers differed in their effects on Mpro functionality. The SAVLQ linker is specifically recognized and cleaved by Mpro (Fig. 3B), which resulted in autocatalytic release of the protease with its native structure and full activity. Active Mpro was toxic to yeast, resulting in impaired cell growth (Fig. 3C). In contrast, with the D4K linker, EGFP remains attached to the N-terminus of Mpro (Fig. 3B), which is believed to limit protease activity. As a result, the growth of yeast cells that expressed EGFP-D4K-Mpro was comparable to that of control yeast cells that expressed EGFP alone (Fig. 3C). A control strain with a *GAL1* deletion (to match the genetic background of the other strains) was also included and showed similar results.

To evaluate the effects of Mpro expression under fermentative and respiratory conditions, different carbon sources were used. These included glucose (YPD) and sucrose (YPS)^11^ which promote rapid fermentation and limit mitochondrial activity, as well as glycerol (YPG)^12,13^, a mixture of glycerol and ethanol (YPEG), or lactate (YPL)^14^ which force cells to activate respiratory pathways. This approach enabled us to assess whether the cytotoxic effects of Mpro are more pronounced under conditions that require high mitochondrial activity.

**Fig. 4:**
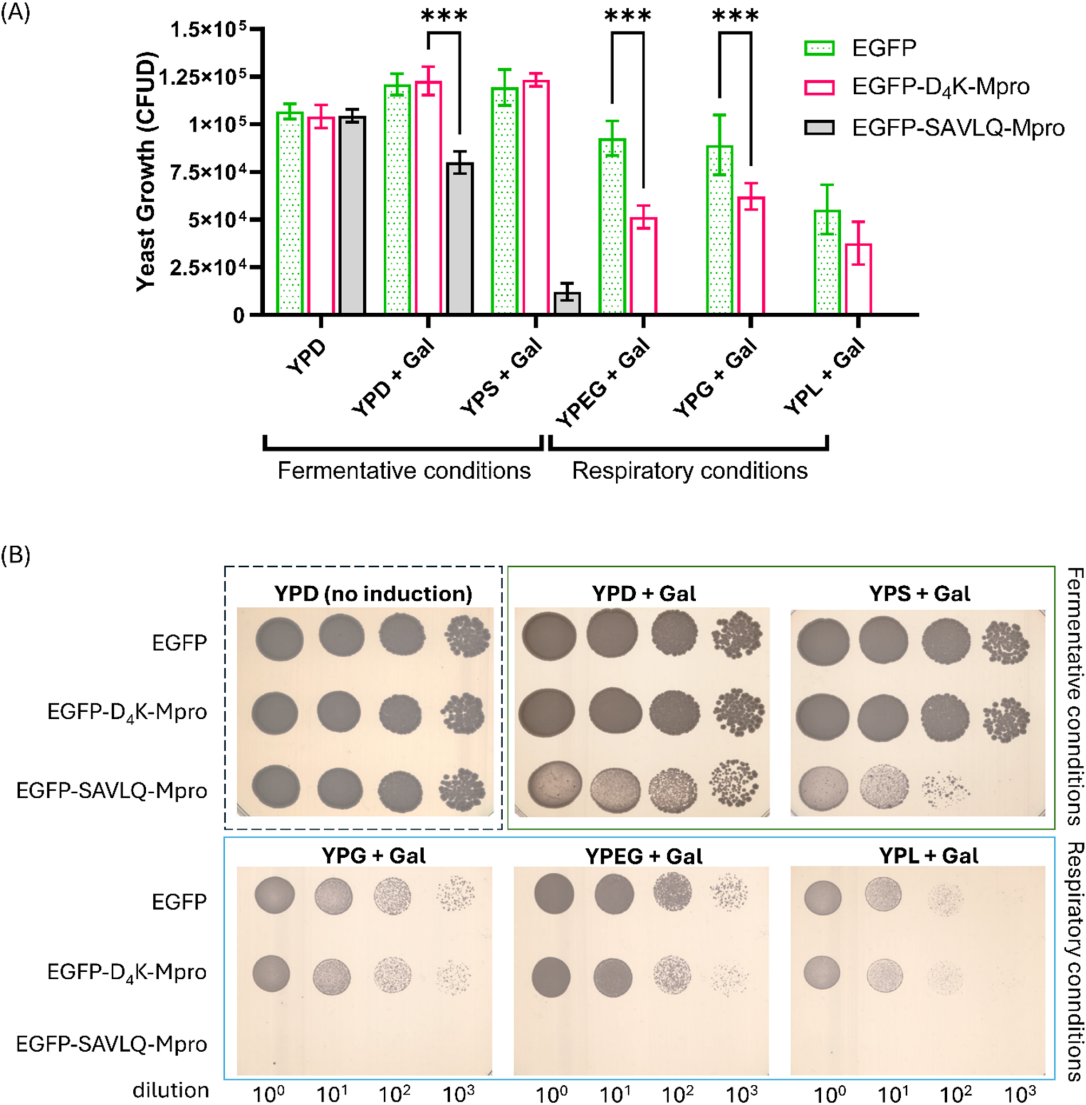
Growth assays of yeast strains under various conditions. (**A**) Differences in yeast growth depending on the carbon source, reflecting yeast metabolism (fermentation or mitochondrial respiration). (**B**) Drop assay results demonstrating the growth of the studied yeast strains in the absence of induction and Mpro gene expression, as controlled by the *GAL1* promoter, and under Mpro gene expression conditions (in the presence of galactose) on media containing different fermentable and nonfermentable carbon sources. Yeast growth was quantified based on colony-forming unit density (CFUD), defined as the total pixel density of colonies from 100-fold serial dilutions. Statistical analysis was performed using two-way ANOVA. Tukey’s multiple comparisons test was used to compare the means within each condition. Statistical analysis was based on three biological replicates. Significant differences between constructs are indicated as ***P < 0.001.

Figure 4 compares the growth of the three strains, allowing a direct assessment of the effect of Mpro expression on yeast cell viability. No toxicity was observed in YPD medium without galactose (Fig. 4A). There were no significant differences between the strains (EGFP, EGFP-SAVLQ-Mpro and EGFP-D4K-Mpro); thus, the introduction of the construct alone did not have a negative effect on the cells.

Under strictly fermentative conditions (YPD + Gal or YPS + Gal), the growth of the EGFP-SAVLQ-Mpro strain was significantly reduced compared with that of both the EGFP control strain and the EGFP-D4K-Mpro variant. This difference was pronounced after induction with galactose in both YPD and YPS, suggesting that active Mpro (formed by the cleavage of the SAVLQ linker) negatively affects cell metabolism under predominantly fermentative conditions. Strong catabolic repression in YPD leads to the discrepancy between the effect in YPD and YPS.

Even more pronounced toxicity of Mpro was observed under conditions requiring intensive cellular respiration (YPG, YPEG and YPL media). The growth of the strain with EGFP-SAVLQ-Mpro often decreased by several orders of magnitude compared with that of the control, clearly indicating that Mpro most strongly interferes with the growth of cells that use mitochondrial metabolism as their main energy source (Fig. 4A). This relationship is perfectly illustrated in Fig. 4B, which shows that no colonies of the strain with SAVLQ were observed on plates supplemented with galactose, whereas the strains expressing EGFP alone or EGFP-D4K-Mpro formed numerous and well-formed colonies.

Interestingly, the expression of the EGFP-D₄K-Mpro construct also significantly affected yeast growth under respiratory conditions, although not as severely as the SAVLQ variant did. The data presented in Fig. 4A suggest that EGFP-D₄K-Mpro partially retained the activity of Mpro, which can disrupt cellular processes, particularly those related to mitochondrial function. As a result, although the toxic effect was not as drastic as that of the SAVLQ variant (in which the protease is fully activated), a significant reduction in colony formation could still be observed under conditions when cells depended on aerobic respiration as their primary source of ATP, ultimately leading to reduced viability and growth of yeast cells.

The results described herein indicate that the presence of SAVLQ in the construct, which is specifically recognized by Mpro, causes full protease activation and inhibition of yeast cell growth, especially when mitochondrial pathways play a dominant role in energy transduction.

### Active Mpro causes mitochondrial dysfunction

**Fig. 5:**
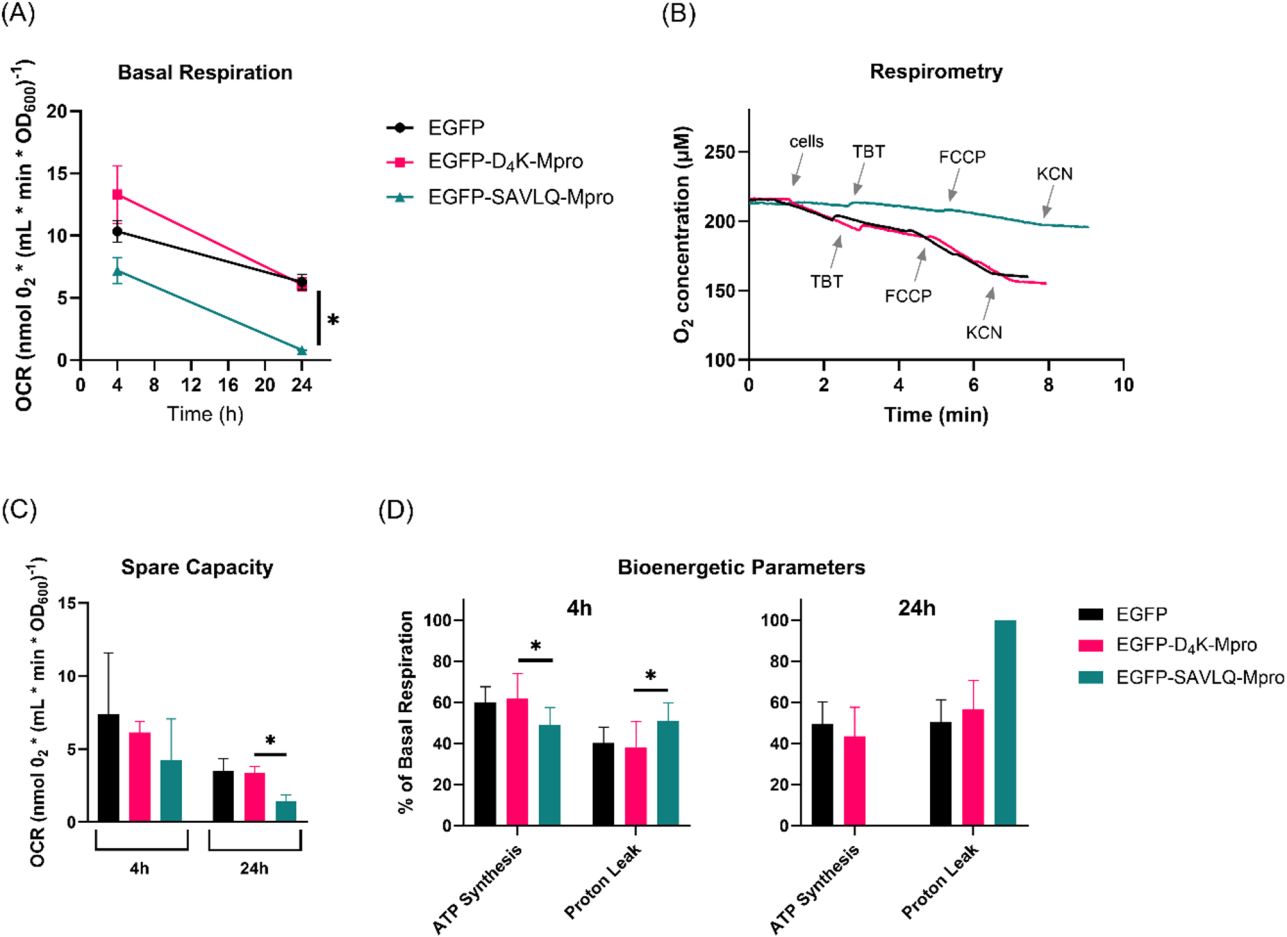
Effects of Mpro on cellular respiration. (**A**) Oxygen consumption rate (OCR) by the studied yeast strains expressing active (EGFP-SALQ-Mpro), less active (EGFP-D4K-Mpro) and EGFP alone, demonstrating the effect of Mpro activity on basal respiration rate. (**B**) Representative respirometry traces showing oxygen consumption responses to sequential additions of metabolic modulators: tributyltin (TBT, an ATP synthase inhibitor), carbonyl cyanide-4-(trifluoromethoxy)phenylhydrazone (FCCP, an uncoupler), and potassium cyanide (KCN, a cytochrome c oxidase inhibitor) after 24 h of induction. (**C**) Mitochondrial spare respiratory capacity measured after 4 h and 24 h of induction. (**D**) Bioenergetic parameters, including ATP synthesis and proton leak, measured as percentages of basal respiration after 4 h and 24 h of induction. Measurements were performed in YPG medium using ~ 3 × 10^7^ cells (OD_600_ = 1). Statistical significance was assessed using the multiple Mann–Whitney test. Data for 4 h induction are based on two independent biological replicates, each with at least three technical replicates. Data for 24 h induction are based on one biological replicate with at least four technical replicates. Significance levels are indicated as: *P < 0.05.

**Fig. 6:**
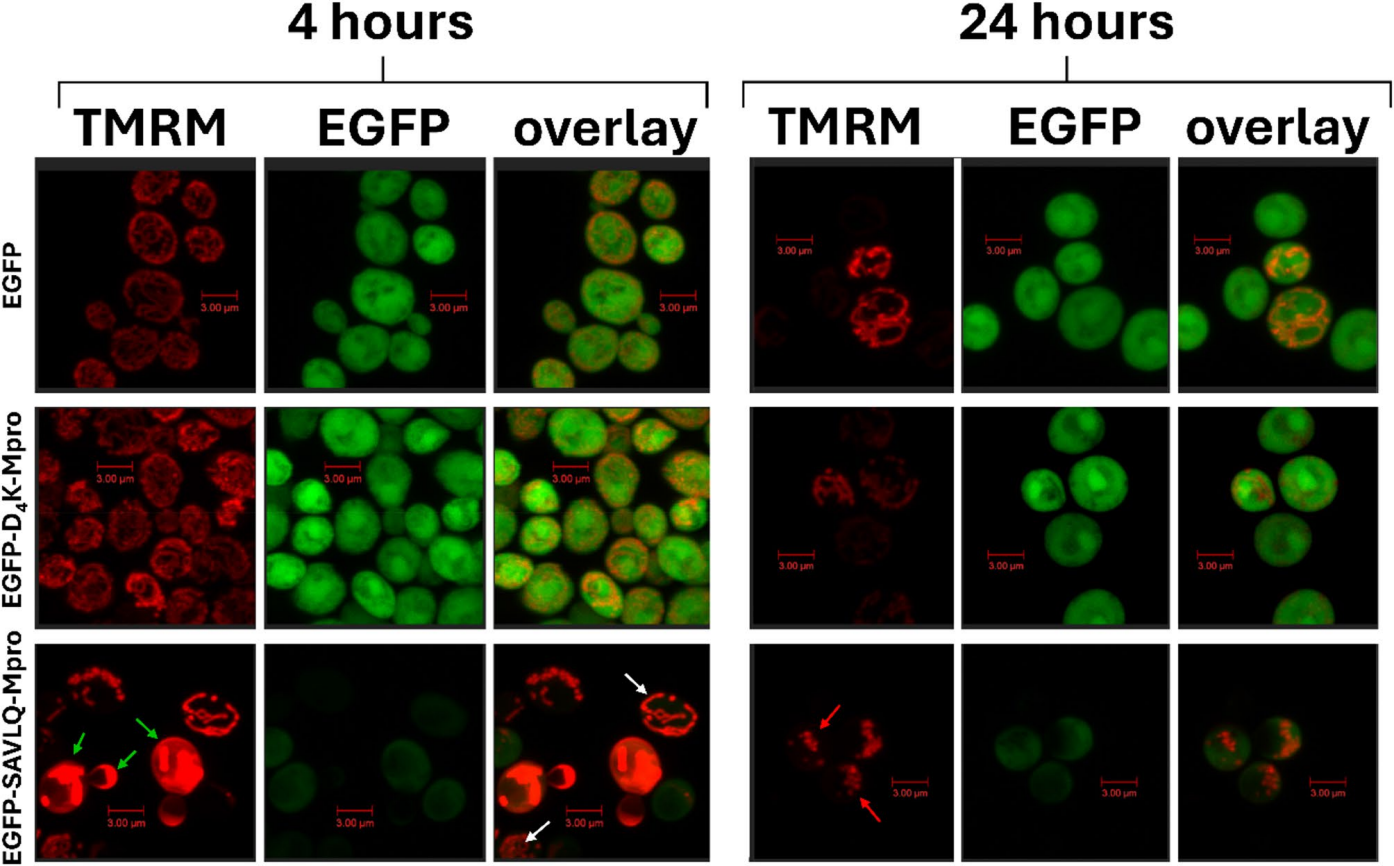
Analysis of the mitochondrial membrane potential and EGFP expression. Confocal microscopy images showing mitochondrial morphology (visualized by TMRM staining, red) and localization of EGFP fusion proteins (green) in yeast strains expressing EGFP alone, EGFP-D₄K-Mpro, and EGFP-SAVLQ-Mpro. Cells were grown in YPG medium with galactose induction. Images were captured after 4 and 24 h of induction. The green arrows indicate areas with abnormalities in mitochondrial morphology, while red arrows highlight aberrant spherical mitochondria. The white arrows indicate mitochondria in cells lacking visible fusion protein expression. Each image shown is representative of at least five independent images captured under identical experimental conditions.

To study the direct effects of active Mpro on mitochondria, a series of experiments were performed to analyze the intensity of cellular respiration and the morphology of the mitochondria. The results are shown in Figs. 5 and 6, which compare three yeast strains grown on YPG medium.

The cellular oxygen consumption rates (OCR) in the presence or absence of mitochondrial respiration modulating substances were assessed in yeast strains expressing either EGFP only (black), EGFP-D4K-Mpro (pink; less active protease) or EGFP-SAVLQ-Mpro (blue; fully active protease) at 4 and 24 h after induction (Fig. 5). The representative result of respirometry measurements after 24 h is presented (Fig. 5B). Compared with the EGFP-SAVLQ-Mpro strain, both EGFP and the EGFP-D4K-Mpro strains presented a significantly greater basal respiration rate, resulting in a more pronounced and rapid decrease in oxygen concentration over time (Fig. 5B). As shown in Fig. 5A basal respiration was significantly reduced in the EGFP-SAVLQ-Mpro strain compared to both EGFP and EGFP-D4K-Mpro strains after 24 h, demonstrating that the presence of the active form of Mpro limits aerobic metabolism in yeast cells.

Additionally, spare respiratory capacity decreased significantly in the EGFP-SAVLQ-Mpro strain (1.43 ± 0.43 nmolO_2_*(ml*min*OD_600_)^−1^) after 24 h compared to the EGFP (3.50 ± 0.84 nmolO_2_*(ml*min*OD_600_)^−1^) and EGFP-D4K-Mpro strains (3.35 ± 0.45 nmolO_2_*(ml*min*OD_600_)^−1^) (Fig. 5C), suggesting reduced metabolic flexibility. Bioenergetic parameter analysis (Fig. 5D) revealed that, after 4 h, the EGFP-D4K-Mpro strain showed elevated ATP-linked respiration (61.73 ± 12.34% of basal respiration) and decreased proton leak (38.27 ± 12.34% of basal respiration) compared to the Mpro-active strain (ATP synthesis 48.93 ± 8.36; proton leak 51.07 ± 8.36% of basal respiration). However, after 24 h proton leak dominated in the EGFP-SAVLQ-Mpro strain, which is consistent with respiratory impairment.

Confocal microscopy was subsequently used to assess mitochondrial morphology, the mitochondrial membrane potential (TMRM dye) and the distribution of the fusion proteins (EGFP signal) in yeast strains EGFP (control), EGFP-D4K-Mpro (less active protease), and EGFP-SAVLQ-Mpro (fully active protease), at 4 and 24 h post-induction (Fig. 6).

In the EGFP and EGFP-D4K-Mpro control strains after 4 h, EGFP expression was evenly distributed and the mitochondria showed intense, homogeneous TMRM staining, indicating intact mitochondrial potential and structure. However, after 24 h some cells in both strains showed a decrease in TMRM staining intensity, reflecting a loss of mitochondrial membrane potential. This is consistent with the observed changes in bioenergetic parameters and spare respiratory capacity measured between 4 and 24 h (Fig. 5C and D).

In contrast, the EGFP-SAVLQ-Mpro strain exhibited abnormal mitochondrial morphology and aberrantly intense accumulation of TMRM (Fig. 6; green arrows) in certain cells already at 4 h, indicative of hyperpolarization. These unusual mitochondrial abnormalities persisted at 24 h. Notably, despite this hyperpolarization, bioenergetic parameters measured did not improve and remained compromised (Fig. 5C and D). These morphological aberrations (Fig. 6; red arrows) observed at 24 h resemble previously described mitochondrial phenotypes associated with defects in essential cellular processes, such as mitochondrial protein import and maintenance of mitochondrial structure, as reported in earlier studies^15^.

Analyses of both the respiratory parameters (Fig. 5) and confocal images (Fig. 6) indicate that the highly active Mpro in yeast can lead to a decrease in respiratory activity and induce mitochondrial malfunction, which is manifested by changes in the membrane potential and alterations in the morphology of these organelles. In contrast, in the variant with an less active form of Mpro (D4K), oxygen metabolism and the mitochondrial structure are preserved, suggesting that when Mpro can self-cleave and be fully activated, it becomes a factor that induces mitochondrial dysfunction. These results provide evidence of the toxic effects of Mpro in yeast cells and further confirm that mitochondria are sensitive targets for the active enzyme.

## Discussion

Our results indicate that Mpro of SARS-CoV-2 is toxic to yeast cells, especially under conditions that require intense mitochondrial activity. We first investigated whether different carbon sources affect the expression levels of genes controlled by the *GAL1* promoter in the yeast system. For this purpose, we constructed a yeast strain expressing free EGFP, which allowed us to verify whether the observed differences were due to the effect of the medium on the activity of the *GAL1* promoter. The results led to the conclusion that certain carbon sources, both fermentable, such as sucrose, and nonfermentable, such as glycerol, were equivalent and yielded comparable expression levels upon use of the inducible expression system based on the *GAL1* promoter.

A major advantage of our system is that it allows use of rich media instead of synthetic media, which are often required for plasmid-based systems to maintain selective pressure. Synthetic media, although well defined, present various challenges, including slower growth rates and metabolic stress, which can confound the results. For example, as highlighted in other studies, yeast growth on glycerol or synthetic media often leads to a long lag phase and requires supplementation with peptone, amino acids, or redox-stabilizing agents, such as acetoin, to improve growth rates and the metabolic balance^16,17^. The medium composition thus significantly influences yeast metabolism and can complicate experimental reproducibility.

The literature indicates that glucose induces repression of the *GAL1* promoter^18^ which was an important reference point in this study. We observed the presence of the EGFP signal even in YPD medium containing galactose, although its intensity was relatively low. This suggests that with the consumption of glucose from the medium, catabolic repression is reversed^19^ and the galactose present in the medium (not metabolized as the yeast lacks the *GAL1* gene, Δ*gal*) acts as an expression inducer. Other carbon sources, such as fermentable sucrose or nonfermentable glycerol, which requires aerobic metabolism, do not interfere with *GAL1* promoter activation, indicating their potential for further study.

A key determinant of the Mpro toxicity was the specific recognition and processing of the SAVLQ linker, which led to the release of the native protease structure. The first N-terminal amino acid, Ser1, stabilizes the active site of the enzyme, which is essential for efficient catalysis^20^. Modifications in this region, such as the addition of GPLGS residues, resulted in a significant decrease in enzymatic activity, whereas changes at the C-terminus were less important for protease function. Although the study focused on Mpro of SARS-CoV-2, the structure of Mpro in SARS-CoV-2 shows considerable similarity to that of its counterpart in SARS-CoV, with the preservation of the key catalytic cysteine‒histidine dyad^21^. Despite minor sequence differences, the mechanism of action of both proteases remains similar^22^. This finding is consistent with the findings of Flynn et al. (2022)^23^ who demonstrated that the restoration of the native N-terminus in a yeast expression system was crucial for the enzymatic activity and associated cellular toxicity of Mpro.

The high expression level and catalytic activity of Mpro, resulting from the reconstitution of its native structure, is most likely the main reason for the observed toxicity of the protease in yeast. The toxicity of Mpro to yeast cells is caused by its ability to nonspecifically cleave endogenous host proteins^24,25^. According to a study by Alalam and colleagues (2021)^25^ Mpro recognizes the sequence [A/S/T]LQ[A/S/G], with 136 potential cleavage sites present in the yeast proteome. Using these data, we identified 23 proteins essential for mitochondrial function that contain potential cleavage sites for Mpro (Table S1).

Similar studies have been performed on human proteins. Predictions indicate that Mpro may interfere with protein trafficking to mitochondria. For example, tRNA methyltransferase 1 (TRMT1) is expected to be cleaved by Mpro, which would result in the removal of the zinc finger and the nuclear localization signal and lead to exclusive mitochondrial localization of TRMT1^4^. A subtiligase and N-terminomic approach was used to identify human substrates of SARS-CoV-2 proteases (Mpro and PLpro)^26^. Analysis of cell lysates with purified SARS-CoV-2 proteases revealed that among various proteins, mitochondrial proteins were also identified as potential substrates of Mpro and PLpro. These include key proteins associated with mitochondrial ribosomes and enzymes involved in energy metabolism.

A study by Cao et al. (2022)^27^ provided a complementary perspective by using a mammalian cell-based system to study the toxicity and enzymatic activity of Mpro. This approach provided a more physiologically representative environment for studying the effects of Mpro on human cellular processes. The native N-terminal structure of Mpro, specifically Ser1, was shown to be critical for its catalytic activity and associated cellular toxicity, whereas N-terminal modifications, e.g., the addition of methionine, led to a significant reduction in toxicity, highlighting the importance of maintaining the native structure of the enzyme. Interestingly, Mpro expression in host cells correlates with apoptosis. This study does not clearly indicate that mitochondria mediate Mpro-induced apoptosis. Therefore, further research is needed to determine whether mitochondria are directly involved in Mpro-induced apoptosis, as apoptosis is often associated with mitochondrial pathways in general cellular contexts. Previous studies have provided evidence that SARS-CoV-2 affects mitochondrial function in host cells, which may have important implications for the progression of COVID-19. By inhibiting OXPHOS^1^SARS-CoV-2 switches cellular metabolism from mitochondrial respiration to glycolysis and the pentose phosphate pathway, which provide the substrates necessary for viral replication. The expression of nuclear genes that are related to mitochondrial function is severely modified during SARS-CoV-2 infection^28^. SARS-CoV-2 modification of mitochondrial function may also play a key role in the development of symptoms associated with long COVID-19^29^. Persistent structural damage and dysregulation of mitochondrial bioenergetics, resulting in compromised energy production, could underlie symptoms including chronic fatigue, muscle weakness, and cognitive disturbances^29^. Moreover, electron microscopic studies have shown abnormalities in the structure of mitochondria in patients with long COVID-19, suggesting a role for mitochondria in the pathogenesis of this syndrome^30^. Although viral proteins have been previously implicated in modifying host mitochondrial function^1^ our results suggest additional roles for active Mpro.

## Conclusions

In this work, we used a yeast model system to study the effects of the SARS-CoV-2 Mpro, focusing on its effects under both fermentative and respiratory conditions. Our data showed that Mpro severely impaired yeast growth in media that forced cells to rely on mitochondrial respiration. Under these conditions, a yeast strain that expressed active Mpro exhibited reduced oxygen consumption, a changed mitochondrial membrane potential, and obvious abnormalities in mitochondrial morphology. In contrast, a strain that expressed a proteolytically less active Mpro variant remained largely unaffected and retained normal mitochondrial function.

These findings suggest that Mpro targets or disrupts yeast mitochondrial pathways, likely by cleaving host proteins that are critical for respiratory metabolism. The observed toxicity correlated with the reconstitution of the native structure of Mpro, highlighting the importance of the N-terminal region of the protease for its enzymatic function. Our results thus suggest that the SARS-CoV-2 Mpro has a significant negative effect on yeast mitochondrial function, which is consistent with emerging evidence that viral proteases can subvert host energy metabolism.

Given that many aspects of mitochondrial physiology and proteostasis are highly conserved among eukaryotes, our yeast system can serve as a powerful model for studying the toxic effects of Mpro at the molecular level. Future research using this platform may deepen our understanding of how Mpro compromises mitochondrial integrity and function, thereby providing important insights into the pathophysiological mechanisms underlying COVID-19.

## Methods

### Plasmids

The plasmids pML104-GAL1 and pBSK(+)-Mpro(D4K) were constructed as described previously^9^. A codon-optimized gene encoding EGFP flanked by downstream (249 nt) and upstream (253 nt) sequences of *GAL1* (pBSK(+)-EGFP plasmid) was constructed using the In-Fusion Snap Assembly (Takara) method. This procedure involved site-directed mutagenesis with the primers Del_D4KMpro_F (5’-TGTACAAATAAGTATACTTCTTTTTTTTACTTT-3’) and Del_D4KMpro_R (5’-ATACTTATTTGTACAATTCATCCATACCATG-3’), using the pBSK(+)-Mpro(D4K) plasmid as a template.

### Strains

All yeast strains used in this study are derived from the *Saccharomyces cerevisiae* BY4741 background, which was purchased from Euroscarf (Frankfurt, Germany), and are listed in Table 1. The EGFP strain was generated using CRISPR/Cas9 technology with the pML104-GAL1 plasmid. The repair DNA was produced by PCR with the primers GAL1A (5’-ACGAATCAAATTAACAACCATAGGA-3’) and GAL1D (5’-ATGTCAAGAATAGGTATCCAAAACG-3’). The yeast transformation procedure was conducted as described in the Yeastmaker Yeast Transformation System 2 (Takara) user manual for small-scale transformation. The transformed cells were subsequently grown on SD-ura medium at 28 °C. For each application of CRISPR/Cas9, the pML104 vector was removed from yeast cells by culture in SDC + 5-FOA medium.

### Media and cell culture

Yeasts were cultured in various media with different carbon sources to observe growth during fermentation or mitochondrial respiration. The compositions and purposes of these media are detailed in Table 2. All media were supplemented with 1% D-galactose when necessary. For yeast genetic engineering, SD-ura or SDC + 5-FOA medium was used. The solid media were prepared by adding 2% agar.

### Yeast growth assay

All yeast strains were grown in liquid YPD medium. Cultures were adjusted to an OD_600_ = 1, and 10-, 100-, and 1000-fold serial dilutions of the cell suspensions were prepared. Each dilution was then transferred onto solid media plates using the drop plate method. The colony-forming unit density (CFUD) was used to assess the growth of yeast cells under various experimental conditions, following the methodology described in the study by Gałgańska et al. (2010)^31^. The plates were incubated at 28 °C for 3 days, followed by scanning. The images were converted to grayscale, and all colonies from the 100-fold dilution were analyzed densitometrically (the total pixel density) using the ImageJ software version 1.54f (https://imagej.net/).

### Quantitative fluorescence analysis

Yeasts expressing EGFP were grown on various solid media. The cells were harvested from the plates with an inoculation loop and resuspended in water. The suspensions were then adjusted to an OD_600_ = 10. For fluorescence intensity analysis, 100 µL of each cell suspension was transferred to a 96-well flat-bottom plate. EGFP fluorescence (ex. 485 nm, em. 532 nm) was measured using The Spark multimode microplate reader (TECAN) at room temperature.

### In-gel fluorescence detection of EGFP

Cell extracts were prepared by mechanical disruption using glass beads in RIPA buffer (50 mM Tris-HCl pH 7.4, 150 mM NaCl, 1% NP-40, 0.5% sodium deoxycholate, 0.1% SDS) supplemented with 1 mM PMSF. Lysates were centrifuged (15,000 x g, 10 min, 4^o^C), and supernatants were mixed 1:1 with Laemmli buffer containing 2-mercaptoethanol. Samples were incubated at 50^o^C for 5 min (not boiled) to preserve EGFP fluorescence. Proteins were separated on 12% SDS-PAGE at room temperature. After electrophoresis, gels were visualized using a UV transilluminator.

### Cellular respiration measurements

Yeast cells were grown in YPG medium to an OD_600_ = 1. To induce gene expression under the *GAL1* promoter, galactose was added to a final concentration of 1%, and the culture was incubated for 4 h or 24 h. The cells were then washed twice with double-distilled water (ddH_2_O) and resuspended in 100 µL of YPG medium. The cell density was measured at OD_600_. The oxygen uptake rates were then measured for the same number of cells in 1 mL of YPG medium by using a Clark electrode (Hansatech Instruments)^32^.

To determine mitochondrial respiratory parameters, the rates of the following respiratory states were assessed: basal respiration, proton leak, ATP synthesis, and maximal respiration. Basal respiration was measured immediately after adding cells to the incubation chamber. To estimate proton leak, 6 µM tributyltin (TBT), an inhibitor of mitochondrial ATP synthase, was added. ATP-linked respiration was calculated by subtracting the proton leak rate from the basal respiration rate. Maximal respiration was induced by the addition of 2 µM FCCP (carbonyl cyanide p-trifluoromethoxyphenylhydrazone), a mitochondrial uncoupler. At the end of each experiment, 1 mM potassium cyanide (KCN) was added to inhibit cytochrome c oxidase and fully suppress mitochondrial respiration.

Based on these measurements, the following parameters were calculated: (a) spare respiratory capacity, defined as the difference between maximal and basal respiration; (b) ATP synthesis contribution to basal respiration; and (c) proton leak contribution to basal respiration, with the latter two expressed as percentages of basal respiration.

### Staining with TMRM

The method was adapted from a published protocol^33^ in which 5% glucose was replaced with 5% sucrose in the buffer. The cell culture was centrifuged, and the pellet was washed with buffer containing 10 mM HEPES (pH 7.0) and 5% sucrose. After the supernatant was removed, the pellet was resuspended in the same buffer containing 50 nM TMRM (#T668; Thermo Fisher Scientific). The cells were stained in the dark for 15 min. The pellet was then washed twice with the same buffer and resuspended in 1 mL of the solution. Finally, 5 µL of the cell suspension was mixed with 25 µL of buffer and dispensed into glass-bottom wells for imaging (CellView, Greiner Bio-One).

### Microscopy

A Leica Stellaris 8 confocal microscope with a white laser and HyD S detectors was used to obtain EGFP and TMRM fluorescence images of the yeast cells. A Leica PL APO 63x/1.20 W CORR CS2 objective was used. Imaging settings were applied to visualize EGFP and TMRM according to the strain characteristics. Images were obtained at 512 × 512 resolution as z-stacks (pixel resolution 0.07 × 0.07 × 0.36 μm). The images were processed at a resolution of 512 × 512 as z-stacks and then converted to maximum projection images. Maximum intensity projection images were used for illustration.

**Table 1:** List of *Saccharomyces cerevisiae* strains used in this work.

Strain	Genotype	Description	Source
EGFP	*MATa his3*Δ1 *leu2*Δ0 *met15*Δ0 *ura3*Δ0 *pdr5*Δ0::KanMX6 *gal1*Δ0::yEGFP	EGFP expression	This work
EGFP-D4K-Mpro	*MATa his3*Δ1 *leu2*Δ0 *met15*Δ0 *ura3*Δ0 *pdr5*Δ0::KanMX6 *gal1*Δ0::yEGFP_(DDDDK)_yMpro	Contains a linker sequence (D4K) between EGFP and Mpro that is not recognized by Mpro, causing the fusion protein to remain intact and resulting in Mpro with less proteolytic activity. This strain does not exhibit any toxic effects, as shown by unrestricted yeast growth.	^9^
EGFP-SAVLQ-Mpro	*MATa his3*Δ1 *leu2*Δ0 *met15*Δ0 *ura3*Δ0 *pdr5*Δ0::KanMX6 *gal1*Δ0::yEGFP_(SAVLQ)_yMpro	Contains a specific sequence (SAVLQ) between EGFP and Mpro, which is recognized and autocatalytically cleaved by Mpro. This leads to the release of Mpro in its native form, facilitating its proteolytic activity and resulting in a toxic effect, as evidenced by restricted yeast growth.	^9^
*gal1*Δ	*MATa his3*Δ1 *leu2*Δ0 *met15*Δ0 *ura3*Δ0 *pdr5*Δ0::KanMX6 *gal1*Δ0	*GAL1* deletion	^9^

**Table 2:** Compositions and purposes of the media used for yeast cultures.

Medium	Composition	Condition/ Purpose
YPD	1% yeast extract, 2% peptone, 2% D-glucose	Fermentation
YPS	1% yeast extract, 2% peptone, 2% sucrose	Fermentation
YPG	1% yeast extract, 2% peptone, 3% glycerol, pH 5.5	Respiration
YPEG	1% yeast extract, 2% peptone, 3% glycerol, 3% ethanol, pH 5.5	Respiration
YPL	1% yeast extract, 2% peptone, 2% lactic acid, pH 5.5	Respiration
SD-ura	0.67% yeast nitrogen base without amino acids, yeast synthetic drop-out medium supplement without uracil, 2% D-glucose	Genetic engineering
SDC + 5-FOA	0.67% yeast nitrogen base without amino acids, yeast complete synthetic drop-out medium supplement, 1 mg/ml 5-fluoroorotic acid, 2% D-glucose	Genetic engineering

## Electronic supplementary material

Below is the link to the electronic supplementary material.

Supplementary Material 1

## Data Availability

All data supporting the results of this study are available in the manuscript and its supplementary file. Additional raw data, plasmid constructs and yeast strains used in this study are available from the corresponding author upon request.
