# Supplementary material for: The SARS-CoV-2 main protease causes mitochondrial dysfunction in a yeast model
Source: Sci Rep. 2025 Jul 18;15:26106. doi: 10.1038/s41598-025-11993-w (PMC12274422; doi:10.1038/s41598-025-11993-w)
Supplement: Supplementary file 1 — Supplementary Material 1 [file 41598_2025_11993_MOESM1_ESM.docx]

**SARS-CoV-2 main protease causes mitochondrial dysfunction in a yeast model**

Wojciech Grabiński, Anna Kicińska, Karolina Funtowicz, Tomasz Skrzypczak, Andonis Karachitos

**Table S1. Potential mitochondrial targets of Mpro.**

| **#** | **Gene** | **Protein Name** | **Subcellular Location** | **Function** |
| --- | --- | --- | --- | --- |
| 1 | ARG8 | Acetylornithine aminotransferase | Mitochondrial matrix | Catalyzes a reaction in the arginine biosynthesis pathway. ^1^ |
| 2 | COX15 | Heme A synthase | Mitochondrial inner membrane | Catalyzes key steps in heme A synthesis, crucial for cytochrome c oxidase function. ^2^ |
| 3 | CRC1 | Mitochondrial carnitine carrier | Mitochondrial inner membrane | Transports various forms of carnitine, aiding in fatty acid metabolism. ^3^ |
| 4 | DIS3 | Exosome complex exonuclease | Cytoplasm, Mitochondrion, Nucleus | An RNA-processing enzyme with roles in mRNA turnover and RNA surveillance across mitochondria and cytoplasm. ^4,5^ |
| 5 | DRE2 | Fe-S cluster assembly protein | Mitochondrial intermembrane space | Facilitates Fe-S cluster assembly, supporting cytoplasmic Fe-S protein maturation and mitigating oxidative stress. ^6–8^ |
| 6 | FMP23 | Protein FMP23, mitochondrial | Mitochondrion | Potentially involved in regulating mitochondrial iron or copper levels. ^9,10^ |
| 7 | MDM1* | Structural protein | Cytoplasm | Crucial for nuclear and mitochondrial inheritance. ^11^ |
| 8 | MIC27 | MICOS complex subunit | Mitochondrial inner membrane | Part of MICOS complex, maintaining mitochondrial membrane structure. ^12^ |
| 9 | MSW1 | Tryptophan--tRNA ligase | Mitochondrial matrix | Catalyzes tryptophan attachment to tRNA, essential for mitochondrial protein synthesis. ^13^ |
| 10 | MTM1 | Mitochondrial carrier protein | Mitochondrial inner membrane | Activates SOD2 by inserting manganese and responds to iron and calorie restriction. ^14,15^ |
| 11 | OAC1 | Mitochondrial oxaloacetate transport protein | Mitochondrial inner membrane | Exchanges dicarboxylates and sulfur oxoanions across the mitochondrial inner membrane. ^16^ |
| 12 | PET309 | Pentatricopeptide repeat-containing protein | Mitochondrial inner membrane | Supports translation initiation and stability of the COX1 gene transcript. ^17^ |
| 13 | PET8 | Mitochondrial carrier protein | Mitochondrial inner membrane | Transports S-adenosylmethionine, necessary for biotin synthesis and respiration. ^18^ |
| 14 | POR2 | Voltage-dependent anion-selective channel protein | Mitochondrial outer membrane | Facilitates ion transport through the outer mitochondrial membrane, with selective permeability based on membrane potential. ^19^ |
| 15 | RIM2 | Mitochondrial carrier protein | Mitochondrial inner membrane | Acts as a carrier protein within the mitochondria. ^20^ |
| 16 | RML2 | Large ribosomal subunit protein uL2m | Mitochondrion | A component of mitoribosomes, aiding in mitochondrial protein synthesis and integration into membranes. ^21^ |
| 17 | SLS1 | Sigma-like sequence protein | Mitochondrial inner membrane | Essential for respiratory chain assembly and mitochondrial gene expression. ^22,23^ |
| 18 | SSC1 | Import motor subunit | Mitochondrial matrix | Drives translocation of proteins into the mitochondrial matrix in an ATP-dependent process. ^24,25^ |
| 19 | SUV3 | ATP-dependent RNA helicase | Mitochondrial matrix | Processes mitochondrial RNA, crucial for RNA turnover and nuclear-mitochondrial interactions. ^26^ |
| 20 | TCM62 | Mitochondrial chaperone | Mitochondrial inner membrane | Assists in assembling succinate dehydrogenase subunits and protects mitochondrial gene expression at high temperatures by preventing ribosomal subunit aggregation. ^27^ |
| 21 | TOM40 | Mitochondrial import receptor subunit | Mitochondrial outer membrane | Forms channels for importing precursor proteins into mitochondria. ^28^ |
| 22 | YEA6 | Mitochondrial NAD transporter | Mitochondrial inner membrane | Transports NAD+ into mitochondria, exchanging with various compounds. ^29,30^ |
| 23 | YIA6 | Mitochondrial NAD transporter | Mitochondrial inner membrane | Transports NAD+ into mitochondria, involved in nucleotide exchange processes. ^29,31^ |

* Although primarily localized in the cytoplasm, this protein is essential for mitochondrial inheritance during cell division. Its degradation could indirectly disrupt mitochondrial distribution and function.

**References**

1. Heimberg, H., Boyen, A., Crabeel, M. & Glansdorff, N. Escherichia coli and Saccharomyces cerevisiae acetylornithine aminotransferase: evolutionary relationship with ornithine aminotransferase. *Gene* **90**, 69–78 (1990).

2. Glerum, D. M., Muroff, I., Jin, C. & Tzagoloff, A. COX15 codes for a mitochondrial protein essential for the assembly of yeast cytochrome oxidase. *J Biol Chem* **272**, 19088–19094 (1997).

3. van Roermund, C. W., Hettema, E. H., van den Berg, M., Tabak, H. F. & Wanders, R. J. Molecular characterization of carnitine-dependent transport of acetyl-CoA from peroxisomes to mitochondria in Saccharomyces cerevisiae and identification of a plasma membrane carnitine transporter, Agp2p. *EMBO J* **18**, 5843–5852 (1999).

4. Dziembowski, A., Lorentzen, E., Conti, E. & Séraphin, B. A single subunit, Dis3, is essentially responsible for yeast exosome core activity. *Nat Struct Mol Biol* **14**, 15–22 (2007).

5. Noguchi, E. *et al.* Dis3, implicated in mitotic control, binds directly to Ran and enhances the GEF activity of RCC1. *EMBO J* **15**, 5595–5605 (1996).

6. Zhang, Y. *et al.* Dre2, a conserved eukaryotic Fe/S cluster protein, functions in cytosolic Fe/S protein biogenesis. *Mol Cell Biol* **28**, 5569–5582 (2008).

7. Vernis, L. *et al.* A newly identified essential complex, Dre2-Tah18, controls mitochondria integrity and cell death after oxidative stress in yeast. *PLoS One* **4**, e4376 (2009).

8. Pandey, A. K., Pain, J., Dancis, A. & Pain, D. Mitochondria export iron-sulfur and sulfur intermediates to the cytoplasm for iron-sulfur cluster assembly and tRNA thiolation in yeast. *J Biol Chem* **294**, 9489–9502 (2019).

9. De Freitas, J. M. *et al.* Exploratory and confirmatory gene expression profiling of mac1Delta. *J Biol Chem* **279**, 4450–4458 (2004).

10. Reinders, J., Zahedi, R. P., Pfanner, N., Meisinger, C. & Sickmann, A. Toward the complete yeast mitochondrial proteome: multidimensional separation techniques for mitochondrial proteomics. *J Proteome Res* **5**, 1543–1554 (2006).

11. McConnell, S. J. & Yaffe, M. P. Nuclear and mitochondrial inheritance in yeast depends on novel cytoplasmic structures defined by the MDM1 protein. *J Cell Biol* **118**, 385–395 (1992).

12. von der Malsburg, K. *et al.* Dual role of mitofilin in mitochondrial membrane organization and protein biogenesis. *Dev Cell* **21**, 694–707 (2011).

13. Myers, A. M. & Tzagoloff, A. MSW, a yeast gene coding for mitochondrial tryptophanyl-tRNA synthetase. *J Biol Chem* **260**, 15371–15377 (1985).

14. Luk, E., Carroll, M., Baker, M. & Culotta, V. C. Manganese activation of superoxide dismutase 2 in Saccharomyces cerevisiae requires MTM1, a member of the mitochondrial carrier family. *Proc Natl Acad Sci U S A* **100**, 10353–10357 (2003).

15. Lee, Y.-L. & Lee, C.-K. Transcriptional response according to strength of calorie restriction in Saccharomyces cerevisiae. *Mol Cells* **26**, 299–307 (2008).

16. Marobbio, C. M. T., Giannuzzi, G., Paradies, E., Pierri, C. L. & Palmieri, F. alpha-Isopropylmalate, a leucine biosynthesis intermediate in yeast, is transported by the mitochondrial oxalacetate carrier. *J Biol Chem* **283**, 28445–28453 (2008).

17. Manthey, G. M. & McEwen, J. E. The product of the nuclear gene PET309 is required for translation of mature mRNA and stability or production of intron-containing RNAs derived from the mitochondrial COX1 locus of Saccharomyces cerevisiae. *EMBO J* **14**, 4031–4043 (1995).

18. Marobbio, C. M. T., Agrimi, G., Lasorsa, F. M. & Palmieri, F. Identification and functional reconstitution of yeast mitochondrial carrier for S-adenosylmethionine. *EMBO J* **22**, 5975–5982 (2003).

19. Guardiani, C. *et al.* yVDAC2, the second mitochondrial porin isoform of Saccharomyces cerevisiae. *Biochim Biophys Acta Bioenerg* **1859**, 270–279 (2018).

20. Marobbio, C. M. T., Di Noia, M. A. & Palmieri, F. Identification of a mitochondrial transporter for pyrimidine nucleotides in Saccharomyces cerevisiae: bacterial expression, reconstitution and functional characterization. *Biochem J* **393**, 441–446 (2006).

21. Amunts, A. *et al.* Structure of the yeast mitochondrial large ribosomal subunit. *Science* **343**, 1485–1489 (2014).

22. Rouillard, J. M. *et al.* SLS1, a new Saccharomyces cerevisiae gene involved in mitochondrial metabolism, isolated as a syntheticlethal in association with an SSM4 deletion. *Mol Gen Genet* **252**, 700–708 (1996).

23. Bryan, A. C., Rodeheffer, M. S., Wearn, C. M. & Shadel, G. S. Sls1p is a membrane-bound regulator of transcription-coupled processes involved in Saccharomyces cerevisiae mitochondrial gene expression. *Genetics* **160**, 75–82 (2002).

24. Morishima, N., Nakagawa, K., Yamamoto, E. & Shibata, T. A subunit of yeast site-specific endonuclease SceI is a mitochondrial version of the 70-kDa heat shock protein. *J Biol Chem* **265**, 15189–15197 (1990).

25. Nakagawa, K., Hashikawa, J., Makino, O., Ando, T. & Shibata, T. Subunit structure of a yeast site-specific endodeoxyribonuclease, endo SceI. A study using monoclonal antibodies. *Eur J Biochem* **171**, 23–29 (1988).

26. Dziembowski, A. *et al.* The yeast mitochondrial degradosome. Its composition, interplay between RNA helicase and RNase activities and the role in mitochondrial RNA metabolism. *J Biol Chem* **278**, 1603–1611 (2003).

27. Dibrov, E., Fu, S. & Lemire, B. D. The Saccharomyces cerevisiae TCM62 gene encodes a chaperone necessary for the assembly of the mitochondrial succinate dehydrogenase (complex II). *J Biol Chem* **273**, 32042–32048 (1998).

28. Rapaport, D. & Neupert, W. Biogenesis of Tom40, core component of the TOM complex of mitochondria. *J Cell Biol* **146**, 321–331 (1999).

29. Todisco, S., Agrimi, G., Castegna, A. & Palmieri, F. Identification of the mitochondrial NAD+ transporter in Saccharomyces cerevisiae. *J Biol Chem* **281**, 1524–1531 (2006).

30. Luongo, T. S. *et al.* SLC25A51 is a mammalian mitochondrial NAD+ transporter. *Nature* **588**, 174–179 (2020).

31. Kory, N. *et al.* MCART1/SLC25A51 is required for mitochondrial NAD transport. *Sci Adv* **6**, eabe5310 (2020).
